# Structural roles of lipid molecules in the assembly of plant PSII−LHCII supercomplex

**DOI:** 10.1007/s41048-018-0068-9

**Published:** 2018-09-12

**Authors:** Xin Sheng, Xiuying Liu, Peng Cao, Mei Li, Zhenfeng Liu

**Affiliations:** 1 National Laboratory of Biomacromolecules, CAS Center for Excellence in Biomacromolecules, Institute of Biophysics, Chinese Academy of Sciences, Beijing, 100101 China; 2 University of Chinese Academy of Sciences, Beijing, 100049 China

**Keywords:** Lipid, Photosystem II, Light-harvesting complex II, Membrane protein, Photosynthesis

## Abstract

In plants, photosystem II (PSII) associates with light-harvesting complexes II (LHCII) to form PSII–LHCII supercomplexes. They are multi-subunit supramolecular systems embedded in the thylakoid membrane of chloroplast, functioning as energy-converting and water-splitting machinery powered by light energy. The high-resolution structure of a PSII–LHCII supercomplex, previously solved through cryo-electron microscopy, revealed 34 well-defined lipid molecules per monomer of the homodimeric system. Here we characterize the distribution of lipid-binding sites in plant PSII–LHCII supercomplex and summarize their arrangement pattern within and across the membrane. These lipid molecules have crucial roles in stabilizing the oligomerization interfaces of plant PSII dimer and LHCII trimer. Moreover, they also mediate the interactions among PSII core subunits and contribute to the assembly between peripheral antenna complexes and PSII core. The detailed information of lipid-binding sites within PSII–LHCII supercomplex may serve as a framework for future researches on the functional roles of lipids in plant photosynthesis.

## Introduction

In plants, algae and cyanobacteria, photosystem II (PSII) cooperates with photosystem I (PSI) and cytochrome *b*_6_*f* (Cyt *b*_6_*f*) to carry out the light-driven electron transport process during the energy conversion process in oxygenic photosynthesis (Nelson and Ben-Shem 2004). The two photosystems and Cyt *b*_6_*f* are multi-subunit membrane protein complexes embedded within the thylakoid membrane, and their proper functions rely on the amphipathic lipid bilayer environments of the membrane. Moreover, specific lipid molecules may participate in the assembly of photosynthetic complexes, serving as their intrinsic components essential for the optimal activity and stability of these membrane protein complexes (Kern and Guskov 2011). There are four major types of lipids in the thylakoid membranes of cyanobacteria and plant chloroplast, namely monogalactosyldiacylglycerol (MGDG), digalactosyldiacylglycerol (DGDG), phosphatidylglycerol (PG), and sulfoquinovosyldiacylglycerol (SQDG) (Mizusawa and Wada 2012). Each of these lipids has distinct polar head group and fulfills specific role in the assembly of photosynthetic complexes (Kern and Guskov 2011; Leng *et al.* 2008). Among them, MGDG and DGDG are the bulk lipids of the thylakoid membranes of cyanobacteria and chloroplasts, and provide amphipathic membrane environments to host photosynthetic complexes (Holzl and Dormann 2007; Mizusawa and Wada 2012). As the predominant neutral glycoglycerolipids of thylakoid membranes, they account for approximately 50% and 30% (mol%) of plant thylakoid membrane lipids, respectively (Siegenthaler 1998). While SQDG and PG are less abundant than MGDG and DGDG, they are anionic lipids contributing negative charges on the surface of the thylakoid membrane and important for photoautotrophic growth of cyanobacteria and plants (Frentzen 2004; Sato 2004).

The functions of these four types of lipid molecules have been well studied through genetic and biochemical approaches. MGDG serves to stimulate functional interaction between plant PSII core complexes and the major light-harvesting complexes II (LHCII), presumably by enhancing physical interactions between the two complexes (Fujii *et al.* 2014; Zhou *et al.* 2009). Moreover, it has an essential role in photoprotection during photosynthesis by supporting the activity of violaxanthin de-epoxidase in the xanthophyll cycle (Jahns *et al.* 2009). MGDG is synthesized by MGDG synthase, and there are three isoforms of MGDG synthases in *Arabidopsis thaliana,* namely MGD1, MGD2, and MGD3 (Kobayashi *et al.* 2013). Among them, MGD1 is the dominant isoform for galactolipid synthesis, and the content of MGDG in *Arabidopsis* MGD1 mutant was reduced to 42% compared to the wild type (Jarvis *et al.* 2000). The reduction of MGDG content, through T-DNA insertion in the MGD1 promoter region or an artificial microRNA targeting MGD1, showed a severe defect in thylakoid membrane development and impaired photosynthetic electron transport (Fujii *et al.* 2014; Zhou *et al.* 2009). Lipase treatment of the PSII sample from *Thermosynechococcus vulcanus* led to degradation of half of the total MGDG in the sample and 16% reduction of the oxygen evolution activity of PSII (Leng *et al.* 2008).

DGDG is a bilayer-forming glycolipid which may be responsible for the formation and stabilization of thylakoid membrane (Lee 2000). There are two isoforms of DGDG synthases (DGD1 and DGD2) involved in DGDG synthesis in *Arabidopsis thaliana*. The content of DGDG in the DGD1 mutant of *Arabidopsis thaliana* is reduced by more than 90% and the mutant plant showed severe growth retardation and altered chloroplast structure when compared to the wild type (Kelly *et al.* 2003). DGDG is required for the functional and structural integrity of the oxygen-evolving complex and thermal stability of plant PSII (Reifarth *et al.* 1997). Deficiency of DGDG lowers the thermal stability of the LHCII–PSII-containing macrodomains and PSI complexes (Krumova *et al.* 2010). Moreover, DGDG is also involved in stabilization of plant LHCII trimers and mediate the interactions between adjacent LHCII trimers at the luminal side (Holzl and Dormann 2007; Liu *et al.* 2004). In cyanobacteria, DGDG may be involved in binding of extrinsic proteins to PSII and stabilizing the oxygen-evolving complex (Sakurai *et al.* 2007). The *dgda* mutant of cyanobacteria contains no detectable DGDG, and also shows growth retardation under high-light stress and high temperature (Mizusawa *et al.* 2009a, b). The mutant exhibits increased sensitivity to photoinhibition and it was suggested that DGDG may have an important role in the repair cycle of photosynthetic complexes (Mizusawa *et al.* 2009b).

As the major phospholipid in thylakoid membranes, PG has important roles in various photosynthetic complexes (Jones 2007; Sato 2004; Wada and Murata 2007). Degradation of PG by enzymatic treatment with phospholipase A_2_ or phospholipase C significantly inhibits the photosynthetic electron transport activities (Jordan *et al.* 1983). The PSII sample purified from *Thermosynechococcus vulcanus* has the content of PG decreased by 59% and the oxygen evolution activity reduced by 40% after being treated with phospholipase A_2_ (Leng *et al.* 2008). The *pgsA* (gene encoding a PG phosphate synthase) mutant of *Synechocystis sp*. PCC 6803 is deficient in biosynthesis of PG, could only grow in the presence of exogenously supplied PG, and the photosynthetic oxygen-evolving activity of the mutant cells was reduced by 40% after a 3-day depletion of PG (Hagio *et al.* 2000). Further study indicated that the content of PSII dimer decreased significantly in the *pgsA* mutant grown under high-light condition, indicating that PG is indispensable for maintaining the dimeric state of PSII (Sakurai *et al.* 2003). A previous biochemical study revealed that spinach PSII core dimer dissociated into monomers when it was treated with phospholipase A_2_, and the PG molecules with trans-hexadecanoic fatty acyl chains can induce dimerization of isolated PSII monomers (Kruse *et al.* 2000). Furthermore, PG is also involved in mediating trimerization of LHCII in plants (Liu *et al.* 2004).

The functional role of SQDG in photosynthesis varies among different species. In *Chlamydomonas*, a SQGD-deficient mutant shows slightly reduced growth rate and 32%–46% decrease in PSII activity compared to the wild type (Sato *et al.* 1995). SQDG is involved in maintaining the structural integrity and thermal stability of PSII from *Chlamydomonas* (Sato *et al.* 2003). In *Arabidopsis*, SQGD-deficient *sqd2* mutants seem to have little impact on photosynthesis when compared to the wild type (Essigmann *et al.* 1998). SQDG may be required for photosynthetic electron transport with limited availability of PG in plants (Kobayashi *et al.* 2016). The SQDG requirement for PSII is species-dependent in cyanobacteria. SQDG-null mutant of *Synechocystis* sp. PCC6803 has decreased photosynthetic and PSII activities, whereas the deficiency of SQDG in PCC7942 strain does not affect PSII activity (Aoki *et al.* 2004).

An in-depth understanding on the pivotal functional roles of lipid molecules in photosynthesis can be achieved by solving the structures of photosynthetic complexes at high resolution (better than 3 Å). Accurate assignment of the binding sites and identities of various lipid molecules in the photosynthetic complexes is indispensible for revealing the specific interactions between lipid molecules and proteins/cofactors within the complexes, as exemplified in the high-resolution crystal structures of cyanobacterial PSI (Jordan *et al.* 2001) and PSII (Umena *et al.* 2011), cytochrome *b*_6_*f* complex (Hasan and Cramer 2014), plant LHCII (Liu *et al.* 2004), and plant PSI (Mazor *et al.* 2017). Previously, crystal structure of a cyanobacterial PSII from *Thermosynechococcus elongatus* (TePSII) was solved at 2.9 Å (Guskov *et al.* 2009) and revealed the presence of 25 lipid molecules per monomer of the PSII homodimer, including 11 MGDG, seven DGDG, five SQDG, and two PG molecules (Kern and Guskov 2011). These lipid molecules serve as multifunctional cofactors involved in the assembly and functional regulation of PSII (Mizusawa and Wada 2012). The high-resolution structure of PSII from *T. vulcanus* (TvPSII) at 1.9 Å contains 20 lipid molecules in each monomer, including six MGDG, five DGDG, four SQDG, and five PG molecules (Umena *et al.* 2011). While all DGDG and SQDG binding sites found in TvPSII are conserved in TePSII, TvPSII contains more PG but less MGDG binding sites than TePSII. As for plant PSII, its core complex exhibits high similarity with cyanobacterial PSII, and it is assembled with peripheral light-harvesting complexes (LHCII, CP29, CP26, and CP24) to form PSII–LHCII supercomplexes (Su *et al.* 2017; Wei *et al.* 2016). Recently, the structures of C_2_S_2_ and C_2_S_2_M_2_-type (C: core; S: strongly associated LHCII; M: moderately associated LHCII) PSII–LHCII supercomplexes have been solved through single-particle cryo-electron microscopy at overall resolutions of 3.2 and 2.7 Å, respectively (Su *et al.* 2017; Wei *et al.* 2016). The assembly between peripheral antennae LHCII/CP29/CP26/CP24 and PSII core relies on the specific interactions between adjacent complexes. Besides protein and pigment molecules, numerous lipid molecules have been located in each monomer of the C_2_S_2_M_2_-type PSII–LHCII supercomplexes. In this review, we focus on discussing the structural roles of lipid molecules in the C_2_S_2_M_2_-type PSII–LHCII supercomplex basing on the 2.7-Å resolution structure and compare them with those found in cyanobacterial and red algal PSII. We have also performed a detailed analysis on the lipid-binding sites in the supercomplex and explain their roles in the assembly of individual complexes and the formation of supercomplex.

## Toward a high-resolution structure of plant PSII–LHCII supercomplexes

Back in 1990s, Boekema *et al.* applied single-particle electron microscopy method to observe the negatively stained sample of spinach PSII and obtained two-dimensional (2D) projection images of the PSII complexes at 15–26 Å resolution (Boekema *et al.* 1995). It was proposed that two LHCII trimers are linked to PSII complex through CP29, CP26, and CP24 to form dimeric PSII–LHCII supercomplex. Subsequently, they were able to identify two different types of LHCII trimers (strongly and moderately associated LHCII, S and M) being associated with PSII core (C), and classify the PSII–LHCII supercomplexes as C_2_S, C_2_S_2_, C_2_SM, C_2_S_2_M and C_2_S_2_M_2_ types using the negatively stained sample (Boekema *et al.* 1998). In 2009, Caffarri *et al.* optimized the purification method for isolating the different classes of *Arabidopsis* PSII–LHCII supercomplexes through sucrose density gradient ultracentrifugation method and improved the 2D projection map of C_2_S_2_M_2_ supercomplex to 12-Å resolution (Caffarri *et al.* 2009). The approximate locations of S-LHCII, M-LHCII, CP29, CP26, and CP24 around the PSII core were assigned in the 2D map.

As the peripheral antenna complexes are weakly associated with plant PSII core complex, the PSII–LHCII supercomplexes are highly unstable and heterogeneous when they are extracted from the membrane and purified in detergent solution. Such a property is unfavorable for growing high-quality three-dimensional (3D) crystal samples. Thus, the attempts to solve the 3D structure of PSII–LHCII supercomplex through X-ray crystallography were unsuccessful, despite that crystals of the reaction center complex of spinach PSII were obtained in the presence of detergent mixtures (Adir 1999). On the other hand, progresses have been made through electron crystallography and single-particle cryo-electron microscopy (cryo-EM) in solving the 3D structures of plant PSII and PSII–LHCII supercomplexes. Rhee *et al.* reported an 8-Å structure of a spinach PSII core complex solved through electron crystallography and assigned the locations of D1, D2 and CP47 subunits (Rhee *et al.* 1997, 1998). Subsequently, Hankamer *et al.* applied the method to solve the structure of spinach PSII core dimer at ~10-Å resolution, found that CP43 and CP47 are located on opposite sides of the central D1–D2 heterodimer and further located the transmembrane helices of major subunits and low-molecular-weight subunits (Hankamer *et al.* 1999, 2001). In 2000, Nield *et al.* reported a 3D model of spinach C_2_S_2_ PSII–LHCII supercomplex basing on a 24-Å map obtained through single-particle cryo-EM method (Nield *et al.* 2000), and further improved the resolution to 17 Å after refinement (Nield and Barber 2006). The approximate locations of PSII core proteins and peripheral antenna complexes were assigned in the low-resolution 3D map, but the detailed features at the interfaces between adjacent complexes remained unknown.

The first near-atomic resolution (3.2 Å) structure of plant PSII–LHCII supercomplex was solved by Wei *et al.* through single-particle cryo-EM method in 2016 (Wei *et al.* 2016). The structure provides detailed information for the locations of most amino acid residues from 25 protein subunits, 105 chlorophylls, and 28 carotenoid molecules within the spinach C_2_S_2_-type PSII–LHCII supercomplex. The specific interactions between adjacent peripheral antenna complexes and between peripheral antenna and core antenna complexes were described in detail, and the potential energy transfer pathways from the antenna complex to the PSII core have been identified (Wei *et al.* 2016). In 2017, Bezouwen *et al.* reported the cryo-EM structure of *Arabidopsis* C_2_S_2_M_2_-type PSII–LHCII supercomplex at 5.3-Å resolution and discussed the subunit and chlorophyll organization within the supercomplex (Bezouwen *et al.* 2017). Furthermore, Su *et al.* solved the structures of stacked and unstacked forms of C_2_S_2_M_2_-type PSII–LHCII supercomplex from *Pisum sativum* (PsPSII–LHCII) at 2.7 and 3.2 Å respectively, and revealed near-atomic details of the protein subunits and various cofactors within the supercomplex (Su *et al.* 2017).

## Overall architecture of PSII–LHCII supercomplex

**Fig. 1:**
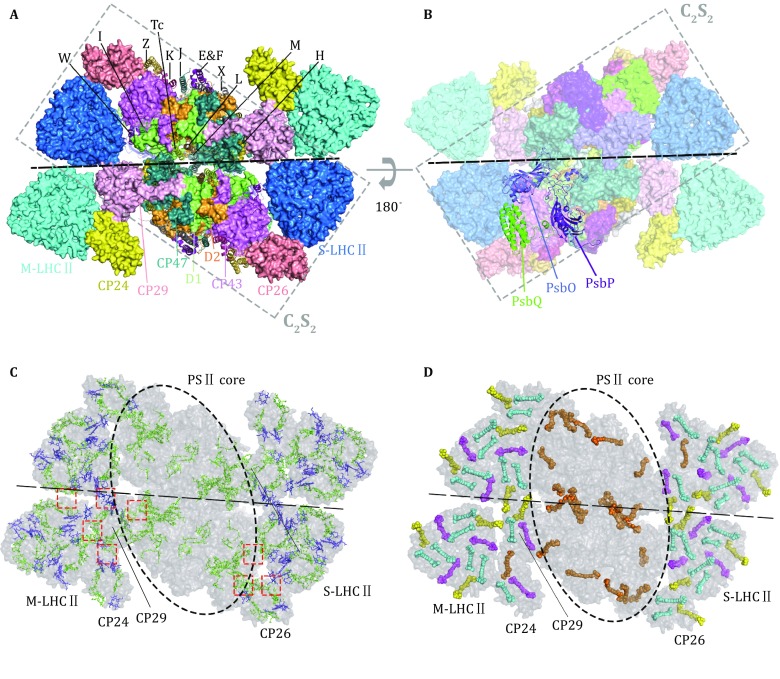
Overall structure of the C_2_S_2_M_2_-type PSII–LHCII supercomplex from *Pisum sativum*. **A, B** The C_2_S_2_M_2_ PSII–LHCII supercomplex viewed from stromal side (**A**) and luminal side (**B**) along the membrane normal. The small intrinsic subunits (labeled by the single-letter or two-letter codes) and extrinsic subunits are presented as cartoon models, while the other subunits are shown as surface models. PDB code: 5XNL. **C, D** Arrangment of chlorophyll (**C**) and carotenoid (**D**) molecules in the C_2_S_2_M_2_ PSII–LHCII supercomplex. The red dashed boxes indicate the interfaces between adjacent peripheral complexes and between peripheral and core complexes. The chlorophyll and carotenoid molecules are presented as stick and sphere models, respectively. Color codes: green, Chl a; blue, Chl b; orange, β-carotene; cyan, lutein; magenta, violaxanthin; yellow, neoxanthin

In the 2.7-Å resolution structure of plant C_2_S_2_M_2_ supercomplex, there are 28 protein subunits, 157 chlorophyll, two pheophytin, 44 carotenoid, one Mn_4_CaO_5_ cluster, 34 lipids, and numerous other cofactors in each monomer. The core complex includes four large intrinsic membrane proteins (D1, D2, CP43, and CP47), 12 small intrinsic proteins (PsbE, PsbF, PsbH, PsbI, PsbJ, PsbK, PsbL, PsbM, PsbTc, PsbW, PsbX, and PsbZ) and three extrinsic proteins (PsbO, PsbP, and PsbQ) on the luminal side (Fig. 1A, B). In the C_2_S_2_ region of the supercomplex, there are strongly associated LHCII trimer and a CP26 monomer attached to two different sides of the CP43 complex, and a CP29 monomer bound to CP47 in the core complex from the other side (Wei *et al.* 2016). To form the C_2_S_2_M_2_ supercomplex, the C_2_S_2_ complex is further assembled with two moderately associated LHCII trimers (M-LHCII) and two CP24 monomers on the sides nearby S-LHCII and CP29 (Fig. 1A). The M-LHCII binds to the concaved groove between S-LHCII and CP29, and also forms extensive interactions with CP24. Furthermore, CP24 interacts closely with CP29 to form a heterodimer, and it is separated from CP47 by a large (~ 30 × 55 Å) void region potentially filled by lipid molecules or unobserved subunits of PSII. The M-LHCII-CP24 subcomplex exhibits high mobility and appears to adopt different tilt positions in the stacked and unstacked C_2_S_2_M_2_ structures (Su *et al.* 2017). The observed off-plane tilting of the subcomplex may facilitate its detachment from the C_2_S_2_ supercomplex and provide a means for the regulation of light harvesting in response to the increase of light intensity (Su *et al.* 2017).

Chlorophyll molecules are the major light-harvesting pigments and there are 108 chlorophyll *a* (Chl *a*) and 49 chlorophyll *b* (Chl *b*) molecules in the structure of C_2_S_2_M_2_ supercomplex. Among them, Chl *a* molecules are widely distributed in both PSII core complexes (CP47, CP43, D1, and D2) and the peripheral antenna complexes (LHCII, CP29, CP26, and CP24). On the other hand, Chl *b* molecules exist only in the peripheral antenna complexes but not in the core complexes (Fig. 1C). At the interfaces between adjacent antenna complexes, there are numerous pairs of chlorophyll molecules (Mg-to-Mg distance at 13–25 Å) forming the potential energy transfer pathways from the peripheral antenna complexes to the core complexes of PSII (Fig. 1C). Besides chlorophylls, carotenoid molecules have important functions in photoprotection, maintain the structural stability of photosynthetic complexes, and may also contribute to light harvesting (Hashimoto *et al.* 2016). There are 44 carotenoid molecules in the C_2_S_2_M_2_ supercomplex, and among them, lutein, violaxanthin and neoxanthin only exist in the peripheral antenna domains. In contrast, β-carotene mainly exists in the PSII core region and there might be one located in CP24 complex (Fig. 1D) (Su *et al.* 2017). These carotenoid molecules are mostly distributed in regions enriched with chlorophylls and form close interactions with them so as to fulfill the photoprotective function during photosynthesis.

## Distribution of lipid-binding sites in the PSII–LHCII supercomplex

**Fig. 2:**
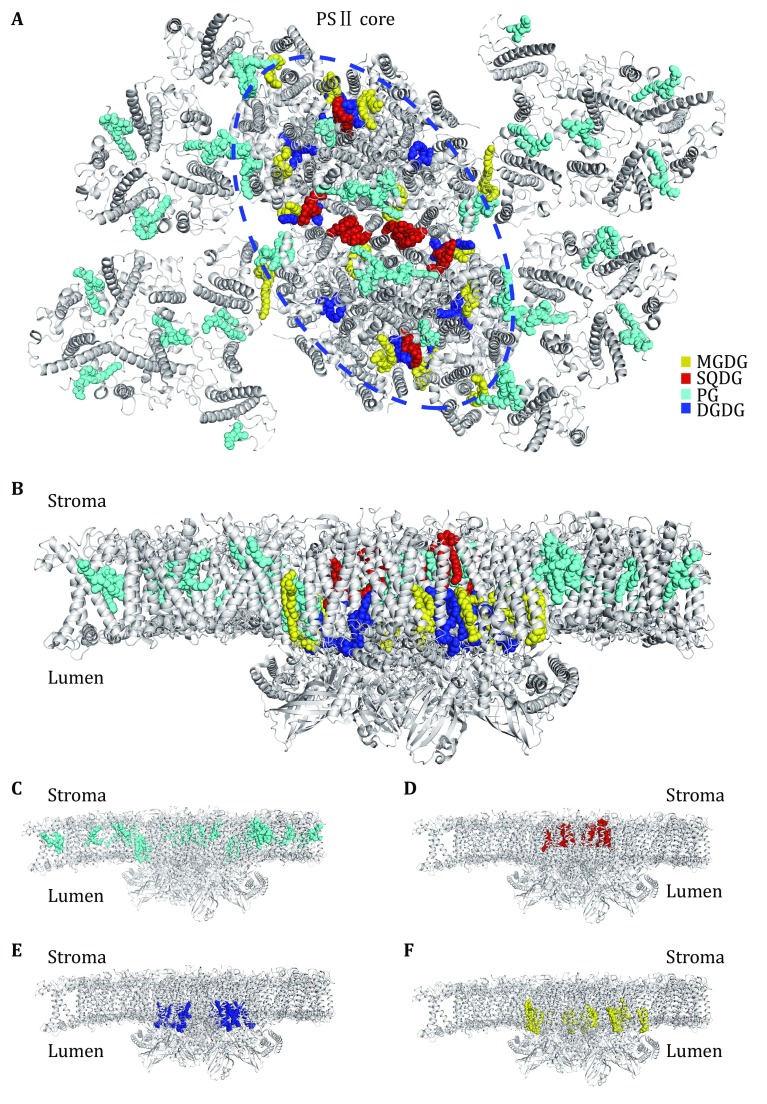
Overall distribution of lipid-binding sites within the C_2_S_2_M_2_ PSII–LHCII supercomplex from *Pisum sativum.* **A** Top view of the supercomplex from the stromal side. The lipid molecules located within the supercomplex are highlighted as colored sphere models, while the proteins are shown as silver cartoon models. The pigments and other cofactors are omitted for clarity. **B** Side view of the supercomplex along the membrane plane. **C**–**F** Asymmetric distributions of PG (**C**), SQDG (**D**), DGDG (**E**) and MGDG (**F**) across the membrane-embedded region within the supercomplex

Within the C_2_S_2_M_2_-type PSII–LHCII supercomplex, the lipid molecules located in each monomer include 18 PG, seven MGDG, five DGDG, and four SQDG (Fig. 2A). Among them, PG molecules are distributed more or less evenly throughout the supercomplex. They are found in the peripheral antenna complexes (LHCII, CP29, CP26 and CP24), at the interfaces between LHCII/CP26/CP29 and CP43/CP47, and within the core complex (at the interfaces between core subunits and around the dimerization interface). Meanwhile, MGDG, DGDG, and SQDG molecules are located mostly in the PSII core region. MGDG molecules are widely distributed around CP43, CP47, D1 and D2 subunits, whereas DGDG molecules are concentrated at the inner core region around D1 and D2 subunits. Three of the SQDG molecules line at the dimerization interface of the PSII core, and one is located at a peripheral cavity surrounded by CP43, PsbK, PsbJ, Cyt *b*559 (PsbE and PsbF), and D1. Curiously, the lipid molecules exhibit an evidently asymmetric distribution pattern across the membrane (Fig. 2C–F). For instance, 16 of the PG molecules have their polar head groups located on the stromal side and only two PG molecules are located on the luminal side (at the interface between CP29 and CP47) (Fig. 2C). All DGDG and MGDG molecules have their head groups positioned at the luminal surface (Fig. 2E, F), while the head groups of all four SQDG molecules are on the stromal side (Fig. 2D). Thereby, the enrichment of anionic lipid molecules (PG and SQDG) on the stromal side contributes significant amount of negative charges on the stromal surface of PSII–LHCII supercomplex. Similar asymmetric distribution of lipid molecules was also observed in the crystal structures of cyanobacterial PSII (TvPSII and TePSII) (Umena *et al.* 2011).

## Role of lipid molecules in stabilizing the PSII core assembly

**Fig. 3:**
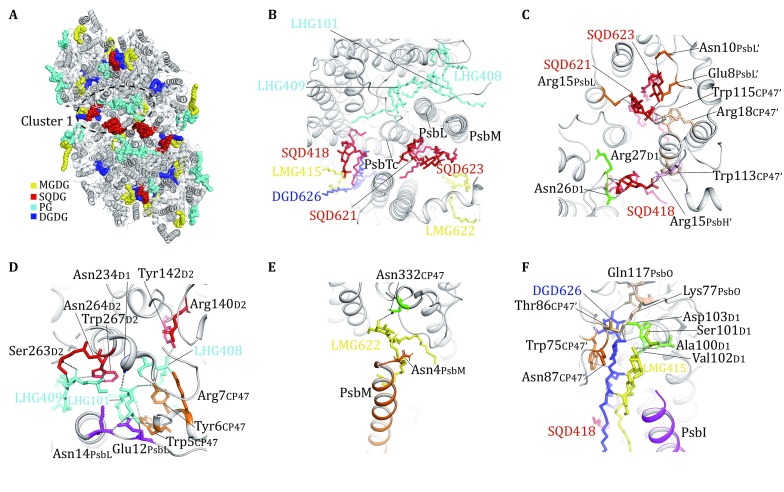
Distribution of lipid-binding sites around the dimerization interface of PsPSII. **A** Top view of the PsPSII core dimer from the stromal side. The lipid molecules are highlighted as colored sphere models. The peripheral antenna complexes (LHCII, CP29, CP26 and CP24) are omitted for clarity. The region around Cluster-1 lipids is labeled by the dashed elliptical ring. **B** Zoom-in view of the Cluster-1 lipid molecules from the stromal side. **C** The binding sites of three SQDG molecules at Cluster 1 region viewed from the stromal side. **D** The binding sites of three PG molecules at Cluster 1 region viewed from the stromal side. **E** The binding sites of a MGDG molecule at Cluster 1 region viewed from luminal side. **F** The binding sites of one MGDG and one DGDG molecule around CP47, D1, PsbO and PsbI subunits viewed from luminal side. The gray dash lines indicate the hydrogen bonds and ionic interactions formed between lipid head groups and nearby amino acid residues. DGD, LHG, LMG and SQD are three-letter codes for DGDG, PG, MGDG and SQDG, respectively

**Fig. 4:**
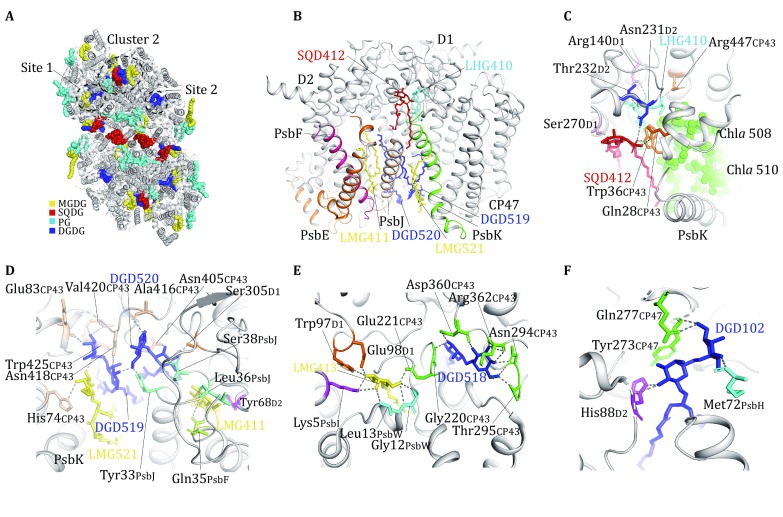
Lipid molecules involved in the assembly of PsPSII core monomer. **A** The locations of Cluster 2, Site 1 and Site 2 lipid-binding regions within the PsPSII core momomer. **B** Lipid molecules at the peripheral Cluster-2 region of the PSII monomer. **C** The binding sites of two lipid molecules (SGD412 and LHG410) surrounded by D1, D2, PsbK and CP43 subunits at stromal side. **D** The binding sites of four lipid molecules (LMG521, DGD519, DGD520, LMG411) at the luminal side of Cluster 2. **E** Two lipid molecules (LMG413 and DGD518) at Site 1 nearby the interface between CP43 and PsbW. **F** A DGDG molecule (DGD102) on Site 2 surrounded by CP47, D2 and PsbH subunits. The hydrogen bonds and ionic interactions between lipid head groups and nearby amino acid residues are shown as dash lines

At the PSII core region of the C_2_S_2_M_2_ supercomplex, the lipid-binding sites are grouped into two clusters (Clusters 1 and 2) and two local binding sites (Sites 1 and 2) buried within CP43 and CP47 (Figs. 3, 4). Cluster 1 is located at the dimerization interface of PSII core, and nine lipid molecules per monomer including three SQDG, three PG, one DGDG and two MGDG are located at this region (Fig. 3A). The PsbTc–PsbL–PsbM trimer is connected with the symmetry-related PsbTc’–PsbL’–PsbM’ trimer through extensive hydrophobic interactions between PsbM and PsbM’. These single-transmembrane-helix small subunits are located at the central dimerization interface of PSII core dimer, and are stabilized by three SQDG (SQD621/SQD623/SQD 418), three PG (LHG101/LHG408/LHG409), one MGDG (LMG622), and their symmetry-related molecules flanking on four sides of the PsbTc–PsbL–PsbM–PsbM’–PsbL’–PsbTc’ hexamer (Fig. 3A, B). On one side, two SQDG molecules (SQD621 and SQD623) are sandwiched between the PsbTc–PsbL–PsbM trimer and the first two transmembrane helices of CP47 from the adjacent monomer of PSII core dimer (CP47′), while the third SQDG (SQD418) is located ~18 Å from SQD621 and intercalates at the space between the first transmembrane helix (M1) of D1 and the second transmembrane helix (M2) of CP47′ (Fig. 3C). The head group of SQD621 forms ionic interactions with Arg15 from PsbL and Arg18 from CP47′ and a hydrogen bond with Trp115_CP47′_, while that of SQD623 is hydrogen-bonded to Asn10 and Glu8 from PsbL’ (Fig. 3C). The head group of SQD418 is hydrogen-bonded to the backbone amide group of Arg27 and side chain of Asn26 from D1 subunit on one side, and forms hydrogen bond with Trp113 from CP47′ and ionic interaction with Arg15 from PsbH’ on the other side. The fatty acyl chains of SQD621, SQD623, SQD418 extend from the stromal surface to the middle region and form hydrophobic interactions with non-polar amino acid residues from adjacent protein subunits, β-carotene molecules and the phytyl chains of chlorophyll molecules located nearby. On the other side, three PG molecules (LHG101, LHG408, and LHG409) are located at the cleft between D1 subunit (the fourth and fifth transmembrane helices/M4 and M5 helices) and PsbTc–PsbL–PsbM trimer (Fig. 3D). The head group of LHG101 is hydrogen-bonded to Asn14 and Glu12 of PsbL, Tyr6 and Trp5 from the N-terminal region of CP47, and Asn234 from D1. The adjacent LHG409 forms hydrogen bonds with Ser263, Trp267, and Asn264 of D2 subunit, while LHG408 on the other side forms hydrogen bond with Tyr6_CP47_ and Tyr142_D2_ as well as ionic interactions with Arg7_CP47_ and Arg140_D2_ (Fig. 3D). Such a tightly packed PG trimer also contributes their fatty acyl chains to bridge the D1 subunit with PsbTc–PsbL–PsbM and CP47 through hydrophobic interactions. Across the membrane, LMG622 (MGDG) has its hydrophobic tails filling in the luminal-side gap between PsbM and the sixth transmembrane helix (M6) of CP47, and its head group forms a hydrogen bond with Asn332_CP47_ and is further bridged to Asn4_PsbM_ through an interstitial water molecule (Fig. 3E).

The remaining two lipid molecules in Cluster 1, namely one DGDG (DGD626) and one MGDG (LMG415), are located on the luminal side in the distal region of the central dimerization interface (Fig. 3F). They are sandwiched between PsbI and M2 of CP47′, and their acyl tails form hydrophobic interactions with the 1-acyl chain of SQD418. The head group of DGDG forms hydrogen bonds with the polar residues from D1 and PsbO simultaneously, and is in van der Waals contact with the side-chain indole ring of Trp75 from CP47′. Therefore, DGD626 is not only involved in dimerization of PSII core, but also has an important role in mediating the assembly of extrinsic protein PsbO with PSII core subunits. The head group of LMG415 is packed closely against that of DGD626, and forms close contact with the N-terminal region of PsbI and Ala100–Val102 region of D1 subunit. On the other side, it faces Thr86–Asn87 region of CP47′ and may be connected to this region through unobserved water molecules.

At the peripheral region of PSII monomer, six lipids molecules (including one SQDG and one PG on the stromal side, two DGDG and two MGDG on the luminal side) are located in the plastoquinone (PQ)–plastoquinol (PQH_2_) exchange cavity near PsbK, PsbJ, and Cyt *b*559, and they form Cluster 2 (Fig. 4A, B). The SQDG (SQD412) and PG (LHG410) molecules form a heterodimer and they are surrounded by the fifth transmembrane helix (M5) of D1 and the fourth transmembrane helix (M4) of D2, PsbK as well as the N-terminal region and the sixth transmembrane helix (M6) of CP43 (Fig. 4C). The head group of SQD412 is hydrogen-bonded to Gln28 and Trp36 from CP43, Ser270 from D1 and Asn231 from D2, while the phospho-(1′-sn-glycerol) head group of LHG410 forms ionic interaction with Arg140 from D1 and hydrogen bonds with Arg447_CP43_, Thr232_D2_, and Asn231_D2_. The fatty acyl chains of SQD412 and LHG410 form extensive hydrophobic interactions with non-polar amino acid residues from D1, PsbK, D2 as well as Chl*a* 508 and Chl*a* 510 from CP43 (Fig. 4C). On the luminal side, the four galactolipids fill in the 24-Å wide void space between the first transmembrane helix of D2 subunit and PsbK, and stabilize the local structure by forming hydrophobic interactions with nearby non-polar groups from D1, D2, PsbF, PsbJ, and PsbK. The digalactosyl head group of DGD519 is hydrogen-bonded to the backbone carbonyl groups of Glu83, backbone amide and carbonyl of Val420, and side chains of Asn418 and Trp425 from CP43, while that of DGD520 forms hydrogen bonds with the backbone carbonyl groups of Ala416_CP43_ and Ser38_PsbJ_ as well as the side chains of Tyr33_PsbJ_, Ser305_D1_, and Asn405_CP43_ (Fig. 4D). The head group of LMG411 is located only 4 Å from that of DGD520, and it is bound to the backbone carbonyl groups of Leu36_PsbJ_ and Tyr68_D2_ as well as the side chain of Gln35_PsbF_ through hydrogen bonds. On the other side, LMG521 is hydrogen-bonded to the head group of DGD519 and His74_CP43_ (Fig. 4D). The hydrophobic fatty acyl chains of the six lipid molecules in Cluster 2, the first three transmembrane helices of D2 subunit, M4 of D1 subunit, Cyt *b*559, PsbJ and PsbK outline a hydrophobic cavity measuring ~22–30 Å wide and 10–22 Å deep. It harbors the PQ molecule on Q_B_ site on one side and opening to the lipid bilayer on the other side through two lateral portals (one is located between PsbE and PsbJ, and the other lies between PsbF and D2). Such a large cavity may serve as a storage pool for more PQ molecules (or lipid molecules) unobserved in the present structure.

Site 1 contains one DGDG (DGD518) and one MGDG (LMG413) on the luminal side and they are located near the fifth transmembrane helix (M5) of CP43, stabilizing the local structure of CP43 and connecting it with D1 and PsbI subunits (Fig. 4A). The head group of DGD518 is hydrogen-bonded to the side chains of Asn294_CP43_ and Thr295_CP43_, backbone carbonyl and amide of Arg362_CP43_, backbone carbonyl of Asp360_CP43_ and backbone amide of Gly220_CP43_ (Fig. 4E). On the other side, LMG413 interacts with DGD518 through its fatty acyl chains, while its head group binds to the side chains of Glu221_CP43_, Trp97_D1_, Glu98_D1_, and Lys5_PsbI_ as well as the backbone amide groups of Leu13_PsbW_ and Gly12_PsbW_ through hydrogen bonds (Fig. 4E). Thus, these two lipid molecules have crucial roles in mediating the assembly among CP43, D1, PsbI, and PsbW. On Site 2, a DGDG molecule (DGD102) at the luminal side binds to a region near the fifth transmembrane helix (M5) of CP47 and fills in the gap among CP47, D2, and PsbH subunits (Fig. 4F). Its digalactosyl head group forms hydrogen bonds with the side chain of Gln277_CP47_ and the backbone carbonyl of Tyr273_CP47_ as well as the side chains of His88_D2_ and Met72_PsbH_ on the other side, while the two fatty acyl chains extend to the hydrophobic interface between CP47 (M5 and M6) and D2 (M2 and M3). Thereby, DGD102 serves to stabilize the assembly among CP47, D2, and PsbH subunits.

## Lipid-binding sites in PSII from different species

**Fig. 5:**
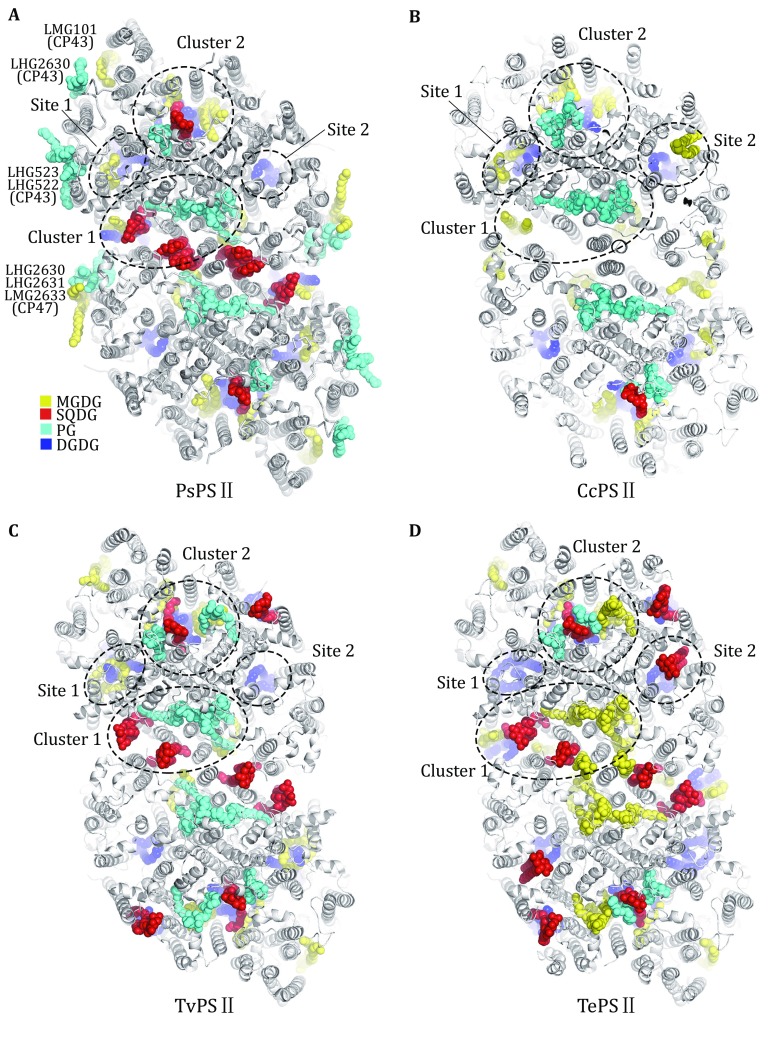
Comparison of the lipid-binding sites in the structures of PSII from four different species. **A**–**D** The core region of PsPSII with the outer antennae omitted (**A**), CcPSII (**B**), TvPSII (**C**) and TePSII (**D**) viewed from the stromal side. Lipid molecules are shown as colored sphere models and protein subunits are presented as cartoon models. Pigment molecules and other cofactors are omitted for clarity. PDB codes: PsPSII, 5XNL; CcPSII, 4YUU; TvPSII, 3WU2; TePSII, 4V62

Currently, there are four structures of PSII from different species with lipid-binding sites assigned at resolutions better than 3-Å resolution, namely PsPSII structure as described in this work, red algal PSII structure from *Cyanidium caldarium* (CcPSII) at 2.76-Å resolution (Ago *et al.* 2016) and two cyanobacterial PSII structures (TvPSII and TePSII). As shown in Fig. 5, PsPSII contains evidently more lipid-binding sites than the red algal and cyanobacterial PSII structures. While the peripheral lipid-binding sites in PsPSII are not present in the other three structures, most the internal sites (Clusters 1 and 2, Sites 1 and 2, shown in Fig. 5A) can be found in CcPSII, TvPSII and TePSII. For the Cluster 1 lipids at the dimerization interface of PsPSII, two SQDG binding sites (SQD418 and SQD621) are also conserved in TvPSII and TePSII, but not found in CcPSII. The third SQDG-binding site (SQD623) at the dimerization interface is occupied by MGDG in TePSII, but is vacant in CcPSII and TvPSII. The three PG-binding sites (LHG101, LHG408 and LHG409) in this cluster are found in CcPSII and TvPSII, but were assigned as MGDG in TePSII. In Cluster 2, the SQD412, LHG410, DGD519, DGD520, LMG411, and LMG521 sites are mostly conserved in PSII from all four different species, while the SQD412 site in CcPSII appears to be occupied by SQDG in one monomer and PG in the other monomer. Meanwhile, TvPSII and TePSII have two additional lipid molecules on the stromal side in the PQ—PQH_2_ exchange cavity area around Cluster 2. The DGD518 and LMG413 of the Site 1 mediate the assembly among CP43, D1, and PsbI in PsPSII, and they are conserved among all four species except that LMG413 site in TePSII is occupied by a DGDG molecule instead of MGDG. The Site-2 lipid DGD102 stabilizes the assembly among CP47, D2, and PsbH, and it is also highly conserved among four species.

In addition to the internal lipid-binding sites, PsPSII contains seven lipid molecules (five PG and two MGDG) at the peripheral regions of CP43 and CP47, namely LMG101, LHG2630, LHG522, and LHG523 around CP43 as well as LHG2630, LHG2631, and LMG2633 around CP47 (Fig. 5A). Their roles in mediating the assembly between CP26/LHCII/CP29 and CP43/CP47 will be discussed in detail in the following section. These lipid molecules are absent in CcPSII or the cyanobacterial PSII since they do not bind the peripheral membrane-embedded antenna complexes as those associate with plant PSII. Instead, cyanobacterial PSII (and CcPSII) associates with an extrinsic light-harvesting apparatus named phycobilisome being attached on the outer/stromal surface of PSII (Chang *et al.* 2015; Zhang *et al.* 2017).

## Locations and roles of lipids in peripheral antenna complexes

**Fig. 6:**
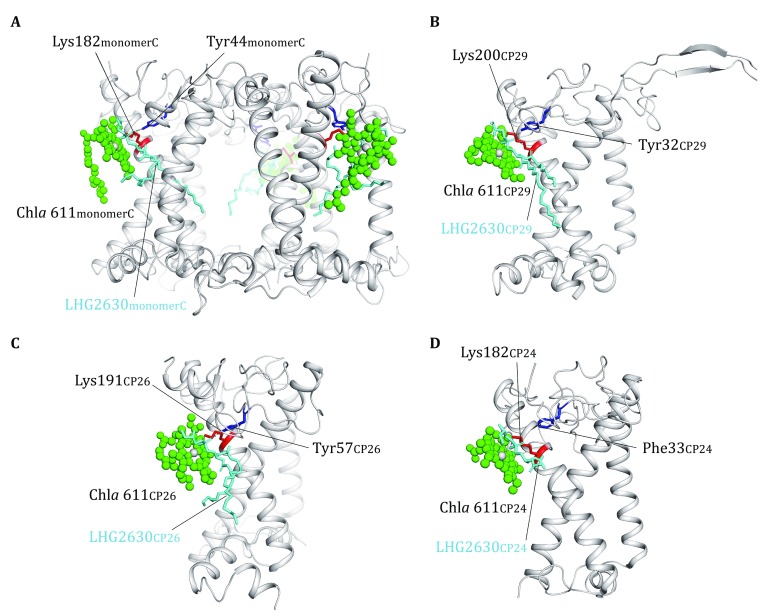
The locations and roles of PG in the peripheral antenna complexes. **A** The role of three PG molecules in the assembly of LHCII trimer. The structure of S-LHCII from the C_2_S_2_M_2_ supercomplex is shown. **B**–**D** The binding sites of PG molecules located in CP29 (**B**), CP26 (**C**) and CP24 (**D**). The chlorophyll molecules coordinated by the phosphate group of PG are presented as green sphere models, and the other pigment molecules are omitted for clarity. The amino acid residues involved in binding the head group of PG are highlighted as colored stick models

In the peripheral antenna complexes (LHCII, CP29, CP26, and CP24), one PG molecule per monomer is located on the stromal side within each complex and it is coordinated with the central Mg atom of Chl *a* 611 in all four complexes. In LHCII, CP29 and CP26, the phosphate group of the Chl *a*-ligating PG molecules is further hydrogen-bonded to the side chains of conserved Tyr residues (Tyr44_LHCII_/Tyr32_CP29_/Tyr57_CP26_) and forms ionic interaction with conserved Lys residues (Lys182_LHCII_/Lys200_CP29_/Lys191_CP26_) (Fig. 6A–C). In CP24, Lys182 forms ionic interaction with the PG phosphate group, whereas the site corresponding to the PG-binding Tyr residues in LHCII/CP29/CP26 is occupied by Phe33 (Fig. 6D). The six PG molecules in S-LHCII and M-LHCII trimers are located at the monomer–monomer interfaces, and mediate trimerization of LHCII (Liu *et al.* 2004). Treatment of LHCII trimers with phospholipase A_2_ cleaved the PG molecules within the complex and led to dissociation of the trimers into monomers (Nussberger *et al.* 1993), demonstrating the important role of PG in stabilizing the LHCII trimer. The PG molecule in CP26 is located at the interface between CP26 and CP43, and mediates the assembly between the two complexes, whereas the PG in CP29 intercalates at the interface between helix A of CP29 and helix C of CP24 and stabilizes the CP29–CP24 heterodimer. The PG molecule in CP24 is located at the peripheral region and may potentially be involved in the assembly of two adjacent C_2_S_2_M_2_ complexes into larger megacomplexes.

## Lipids at the interfaces between LHCII/CP26/CP29 and PSII core

**Fig. 7:**
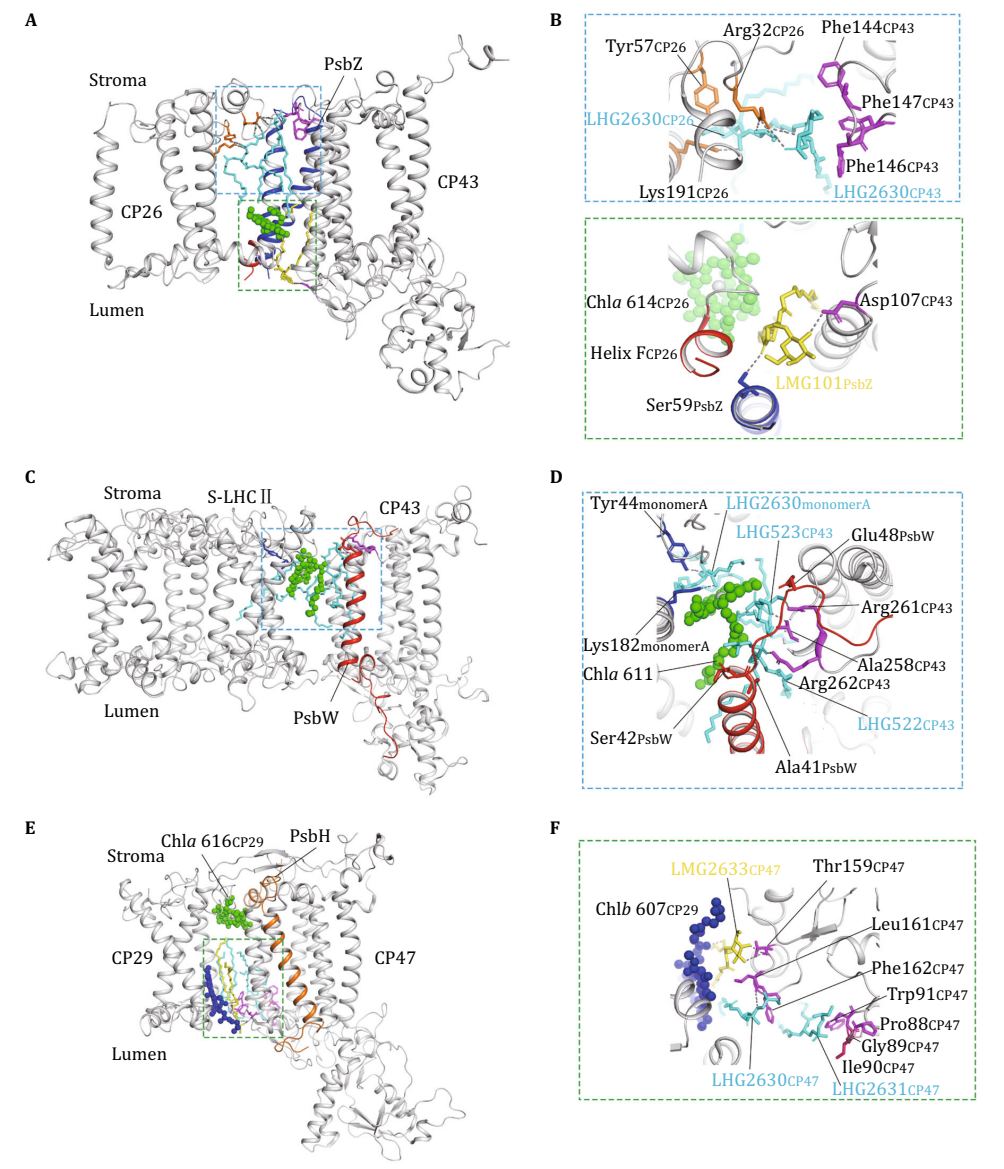
The lipid molecules at the interfaces between LHCII/CP26/CP29 and PSII core complexes. **A** Side view of the interfacial lipid molecules between CP26 and CP43. **B** The zoom-in views of the lipid-binding sites in the blue and green dashed boxes shown in **A**. In the blue dashed box area (upper part), two PG molecules on the stromal side mediate the interactions between CP26 and CP43. In the green dashed box area (lower part), one MGDG molecule is located on the luminal side of the interface between CP26 and CP43. **C** Side view of the interfacial lipid molecules between S-LHCII and CP43. **D** Zoom-in view of the three PG molecules on the stromal side of the interface between S-LHCII and CP43. **E** Side view of the interfacial lipid molecules between CP29 and CP47. **F** Zoom-in view of the three lipid-binding sites on the luminal side of the interface between CP29 and CP47. The lipid molecules and amino acid residues involved in binding the lipid molecules are highlighted as stick models, while the protein backbones are shown as silver cartoon models. The chlorophyll molecules are shown as sphere models

The assembly between S-LHCII/CP26/CP29 and PSII core involves three small intrinsic subunits, namely PsbW, PsbZ, and PsbH, located at the S-LHCII–CP43, CP26–CP43, and CP29–CP47 interfaces, respectively (Wei *et al.* 2016). In addition to these small subunits, there are two PG molecules and one MGDG molecule located at the CP26–CP43 interface (Fig. 7A, B), three PG molecules at the LHCII–CP43 interface (Fig. 7C, D) and two PG plus one MGDG molecules at the CP29–CP47 interface (Fig. 7E, F). On the stromal side of the CP26–CP43 interface, LHG2630_CP26_ and LHG2630_CP43_ form a closely packed lipid dimer (Fig. 7B). In addition to the hydrophobic interactions between the 2-acyl groups of these two PG molecules, the head group glycerol of LHG2630_CP26_ is hydrogen-bonded to the phosphate group of LHG2630_CP43_. While LHG2630_CP26_ binds to Lys191 from Helix A as well as Tyr57 and Arg32 from the N-terminal region of CP26 respectively, LHG2630_CP43_ has its head group surrounded by three phenylalanine residues (Phe144, Phe146, Phe147) from M2–M3 loop region of CP43. The four fatty acyl chains of these two PG molecules fill in the stromal-side gap between M3 helix of CP43 and Helix A of CP26 (Fig. 7B). On the luminal side, LMG101 has its head group linked to Asp107 from CP43 and Ser59 from PsbZ through hydrogen bonds on one side, and forms van der Waals contacts with amino acid residues from the C-terminal Helix F of CP26 (Fig. 7B). The two acyl chains of LMG101 insert in the luminal gap between M2 helix of CP43 and Chl *a*614 of CP26, and connect them through hydrophobic interactions. Thus, these three lipid molecules and PsbZ subunit collectively stabilize the assembly between CP26 and CP43.

At the S-LHCII–CP43 interface, there are three PG molecules (LHG2630_S-LHCII/monomer A_, LHG523_CP43_, and LHG522 _CP43_) on the stromal side (Fig. 7C). The glycerol head group of LHG523 is hydrogen-bonded to the side chain of Glu48 from the C-terminal region of PsbW as well as the backbone amide of Ala258 and Arg261 from the M4–M5 loop region of CP43 (Fig. 7D). For LHG522, its head group glycerol is in van der Waals contact with the backbones of Ala41–Ser42 on the transmembrane helix of PsbW and the phosphate group forms a strong ionic interaction with Arg262 from CP43. LHG522 and LHG523 form a closed packed dimer, and their fatty acyl chains intercalate between the M4 of CP43 and PsbW, and fill in the space between S-LHCII and CP43. LHG523 is further associated with Chl *a*611 of S-LHCII (monomer A) through van der Waals contacts and hydrophobic interactions. On the other side, the head group of LHG2630_S-LHCII/monomer A_ is ligated with the central Mg atom of Chl *a* 611 and connected to Lys182 and Tyr44 through salt bridge and hydrogen bond respectively. While the 2-acyl chain of LHG2630_S-LHCII/monomer A_ contributes to the monomer–monomer interface of the LHCII trimer, its 1-acyl chain bends over toward the interfacial region between S-LHCII and CP43 and makes contact with the 2-acyl chain of LHG523 (Fig. 7D). Therefore, these three PG molecules serve to stabilize the assembly among S-LHCII, PsbW, and CP43.

As for the interface between CP29 and CP47, the stromal-side gap between the two complexes is tightly packed by Chl *a*616 from CP29 and amino acid residues from the N-terminal region of CP29, leaving little space for lipid molecules to bind (Fig. 7E). In addition to direct CP29–CP43 interactions on the stromal side, the N-terminal region of PsbH intercalates between the N-terminal region of CP29 and the stromal surface of CP47 and serves as a bolt securing their interface (Wei *et al.* 2016). On the luminal side, three lipid molecules (LMG2633_CP47_, LHG2630_CP47_, and LHG2631_CP47_) are sandwiched between CP29 and CP47 (Fig. 7E). The galactosyl head group of LMG2633_CP47_ is hydrogen-bonded to the backbone carbonyl of Thr159 _CP47_, and the head group glycerol of LHG2630_CP47_ binds to the backbone carbonyl groups of Phe162 and Leu161 from the M3–M4 loop region of CP47. The head group of LHG2631_CP47_ is in contact with the Pro88–Trp91 region on the M1–M2 loop of CP47 (Fig. 7F). The six fatty acyl chains of these three lipid molecules fill in the space between the M3 helix of CP47 and Helix C of CP29 and the space between the M4 helix of CP47 and Chl *b*607_CP29_ near Helix E of CP29. Thereby, the assembly between CP29 and CP47 is stabilized by three interfacial lipid molecules on the luminal side.

At the M-LHCII–CP29, M-LHCII–S-LHCII, S-LHCII–CP26, S-LHCII–CP47, and CP24–PsbH/PsbX interfaces, there are some apparent void areas between the adjacent complexes. The areas may be filled with more lipid molecules, and they are either lost during purification or too disordered to be observed in the structure.

## Summary and perspectives

The high-resolution structure of plant C_2_S_2_M_2_-type PSII–LHCII supercomplex reveals remarkable structural roles of lipid molecules in stabilizing the PSII core complex. Moreover, they contribute to oligomerization of PSII core dimer and LHCII trimers, and mediate the assembly between PSII and the peripheral antenna complexes including LHCII, CP29, CP26, and CP24. Furthermore, they might influence the biological function of the supercomplex by interacting with the neighboring protein subunits and the function-related cofactors, namely chlorophylls and carotenoids. Curious questions remain open concerning how the interfacial lipid molecules affect the energy transfer and electron transport kinetics as well as the spectroscopic features of the PSII–LHCII supercomplex. In perspective, future investigation on the effect of targeted mutagenesis on the above-mentioned lipid-binding sites (through the CRISPR/Cas9 genome editing technique (Bortesi and Fischer 2015), for instance) is highly anticipated. More specifically, it will be interesting if one could select and mutate the amino acid residues involved in binding specific lipid molecules within the PSII–LHCII supercomplex, and examine the functional behaviors of the mutants. The mechanistic insights obtained will improve our understanding on the roles of individual lipid molecules in the assembly and function of PSII–LHCII supercomplex. To this end, the detailed features of the lipid-binding sites described in this review can serve as a guide for designing new experiments to analyze the functional roles of each individual lipid molecules within the supercomplex.

## References

[CR1] Adir N (1999). Crystallization of the oxygen-evolving reaction centre of photosystem II in nine different detergent mixtures. Acta Crystallogr D Biol Crystallogr.

[CR2] Ago H, Adachi H, Umena Y, Tashiro T, Kawakami K, Kamiya N, Tian L, Han G, Kuang T, Liu Z, Wang F, Zou H, Enami I, Miyano M, Shen JR (2016). Novel features of eukaryotic photosystem II revealed by its crystal structure analysis from a red alga. J Biol Chem.

[CR3] Aoki M, Sato N, Meguro A, Tsuzuki M (2004). Differing involvement of sulfoquinovosyl diacylglycerol in photosystem II in two species of unicellular cyanobacteria. Eur J Biochem.

[CR4] Bezouwen LS, Caffarri S, Kale RS, Kouřil R, Awh T, Oostergetel GT, Boekema EJ (2017). Subunit and chlorophyll organization of the plant photosystem II supercomplex. Nat Plants.

[CR5] Boekema EJ, Hankamer B, Bald D, Kruip J, Nield J, Boonstra AF, Barber J, Rögner M (1995). Supramolecular structure of the photosystem II complex from green plants and cyanobacteria. Proc Natl Acad Sci USA.

[CR6] Boekema EJ, Roon HV, Dekker JP (1998). Specific association of photosystem II and light-harvesting complex II in partially solubilized photosystem II membranes. FEBS Lett.

[CR7] Bortesi L, Fischer R (2015). The CRISPR/Cas9 system for plant genome editing and beyond. Biotechnol Adv.

[CR8] Caffarri S, Kouřil R, Kereïche S, Boekema EJ, Croce R (2009). Functional architecture of higher plant photosystem II supercomplexes. EMBO J.

[CR9] Chang L, Liu X, Li Y, Liu CC, Yang F, Zhao J, Sui SF (2015). Structural organization of an intact phycobilisome and its association with photosystem II. Cell Res.

[CR10] Essigmann B, Guler S, Narang RA, Linke D, Benning C (1998). Phosphate availability affects the thylakoid lipid composition and the expression of SQD1, a gene required for sulfolipid biosynthesis in *Arabidopsis thaliana*. Proc Natl Acad Sci USA.

[CR11] Frentzen M (2004). Phosphatidylglycerol and sulfoquinovosyldiacylglycerol: anionic membrane lipids and phosphate regulation. Curr Opin Plant Biol.

[CR12] Fujii S, Kobayashi K, Nakamura Y, Wada H (2014). Inducible knockdown of MONOGALACTOSYLDIACYLGLYCEROL SYNTHASE1 reveals roles of galactolipids in organelle differentiation in *Arabidopsis cotyledons*. Plant Physiol.

[CR13] Guskov A, Kern J, Gabdulkhakov A, Broser M, Zouni A, Saenger W (2009). Cyanobacterial photosystem II at 2.9 Å resolution and the role of quinones, lipids, channels and chloride. Nat Struct Mol Biol.

[CR14] Hagio M, Gombos Z, Varkonyi Z, Masamoto K, Sato N, Tsuzuki M, Wada H (2000). Direct evidence for requirement of phosphatidylglycerol in photosystem II of photosynthesis. Plant Physiol.

[CR15] Hankamer B, Morris EP, Barber J (1999). Revealing the structure of the oxygen-evolving core dimer of photosystem II by cryoelectron crystallography. Nat Struct Biol.

[CR16] Hankamer B, Morris E, Nield J, Gerle C, Barber J (2001). Three-dimensional structure of the photosystem II core dimer of higher plants determined by electron microscopy. J Struct Biol.

[CR17] Hasan SS, Cramer WA (2014). Internal lipid architecture of the hetero-oligomeric cytochrome *b6f* complex. Structure.

[CR18] Hashimoto H, Uragami C, Cogdell RJ (2016). Carotenoids and photosynthesis. Subcell Biochem.

[CR19] Holzl G, Dormann P (2007). Structure and function of glycoglycerolipids in plants and bacteria. Prog Lipid Res.

[CR20] Jahns P, Latowski D, Strzalka K (2009). Mechanism and regulation of the violaxanthin cycle: the role of antenna proteins and membrane lipids. Biochim Biophys Acta.

[CR21] Jarvis P, Dormann P, Peto CA, Lutes J, Benning C, Chory J (2000). Galactolipid deficiency and abnormal chloroplast development in the *Arabidopsis* MGD synthase 1 mutant. Proc Natl Acad Sci USA.

[CR22] Jones MR (2007). Lipids in photosynthetic reaction centres: structural roles and functional holes. Prog Lipid Res.

[CR23] Jordan BR, Chow WS, Baker AJ (1983). The role of phospholipids in the molecular-organization of pea chloroplast membranes—effect of phospholipid depletion on photosynthetic activities. Biochim Biophys Acta.

[CR24] Jordan P, Fromme P, Witt HT, Klukas O, Saenger W, Krauss N (2001). Three-dimensional structure of cyanobacterial photosystem I at 2.5 Å resolution. Nature.

[CR25] Kelly AA, Froehlich JE, Dormann P (2003). Disruption of the two digalactosyldiacylglycerol synthase genes DGD1 and DGD2 in *Arabidopsis* reveals the existence of an additional enzyme of galactolipid synthesis. Plant Cell.

[CR26] Kern J, Guskov A (2011). Lipids in photosystem I: multifunctional cofactors. J Photochem Photobiol B.

[CR27] Kobayashi K, Narise T, Sonoike K, Hashimoto H, Sato N, Kondo M, Nishimura M, Sato M, Toyooka K, Sugimoto K, Wada H, Masuda T, Ohta H (2013). Role of galactolipid biosynthesis in coordinated development of photosynthetic complexes and thylakoid membranes during chloroplast biogenesis in *Arabidopsis*. Plant J.

[CR28] Kobayashi K, Endo K, Wada H (2016). Roles of lipids in photosynthesis. Subcell Biochem.

[CR29] Krumova SB, Laptenok SP, Kovacs L, Toth T, van Hoek A, Garab G, van Amerongen H (2010). Digalactosyl-diacylglycerol-deficiency lowers the thermal stability of thylakoid membranes. Photosynth Res.

[CR30] Kruse O, Hankamer B, Konczak C, Gerle C, Morris E, Radunz A, Schmid GH, Barber J (2000). Phosphatidylglycerol is involved in the dimerization of photosystem II. J Biol Chem.

[CR31] Lee AG (2000). Membrane lipids: it’s only a phase. Curr Biol.

[CR32] Leng J, Sakurai I, Wada H, Shen JR (2008). Effects of phospholipase and lipase treatments on photosystem II core dimer from a thermophilic cyanobacterium. Photosynth Res.

[CR33] Liu Z, Yan H, Wang K, Kuang T, Zhang J, Gui L, An X, Chang W (2004). Crystal structure of spinach major light-harvesting complex at 2.72 Å resolution. Nature.

[CR34] Mazor Y, Borovikova A, Caspy I, Nelson N (2017). Structure of the plant photosystem I supercomplex at 2.6 Å resolution. Nat Plants.

[CR35] Mizusawa N, Wada H (2012). The role of lipids in photosystem II. Biochim Biophys Acta.

[CR36] Mizusawa N, Sakata S, Sakurai I, Sato N, Wada H (2009). Involvement of digalactosyldiacylglycerol in cellular thermotolerance in *Synechocystis* sp. PCC 6803. Arch Microbiol.

[CR37] Mizusawa N, Sakurai I, Sato N, Wada H (2009). Lack of digalactosyldiacylglycerol increases the sensitivity of *Synechocystis* sp. PCC 6803 to high light stress. FEBS Lett.

[CR38] Nelson N, Ben-Shem A (2004). The complex architecture of oxygenic photosynthesis. Nat Rev Mol Cell Biol.

[CR39] Nield J, Barber J (2006). Refinement of the structural model for the photosystem II supercomplex of higher plants. Biochim Biophys Acta.

[CR40] Nield J, Orlova EV, Morris EP, Gowen B, Van HM, Barber J (2000). 3D map of the plant photosystem II supercomplex obtained by cryoelectron microscopy and single particle analysis. Nat Struct Biol.

[CR41] Nussberger S, Dorr K, Wang DN, Kuhlbrandt W (1993). Lipid-protein interactions in crystals of plant light-harvesting complex. J Mol Biol.

[CR42] Reifarth F, Christen G, Seeliger AG, Dormann P, Benning C, Renger G (1997). Modification of the water oxidizing complex in leaves of the *dgd1* mutant of *Arabidopsis thaliana* deficient in the galactolipid digalactosyldiacylglycerol. Biochemistry.

[CR43] Rhee KH, Morris EP, Zheleva D, Hankamer B, Kuhlbrandt W, Barber J (1997). Two-dimensional structure of plant photosystem II at 8 Å resolution. Nature.

[CR44] Rhee KH, Morris EP, Barber J, Kühlbrandt W (1998). Three-dimensional structure of the plant photosystem II reaction centre at 8 Å resolution. Nature.

[CR45] Sakurai I, Hagio M, Gombos Z, Tyystjarvi T, Paakkarinen V, Aro EM, Wada H (2003). Requirement of phosphatidylglycerol for maintenance of photosynthetic machinery. Plant Physiol.

[CR46] Sakurai I, Mizusawa N, Wada H, Sato N (2007). Digalactosyldiacylglycerol is required for stabilization of the oxygen-evolving complex in photosystem II. Plant Physiol.

[CR47] Sato N (2004). Roles of the acidic lipids sulfoquinovosyl diacylglycerol and phosphatidylglycerol in photosynthesis: their specificity and evolution. J Plant Res.

[CR48] Sato N, Sonoike K, Tsuzuki M, Kawaguchi A (1995). Impaired photosystem II in a mutant of *Chlamydomonas reinhardtii* defective in sulfoquinovosyl diacylglycerol. Eur J Biochem.

[CR49] Sato N, Aoki M, Maru Y, Sonoike K, Minoda A, Tsuzuki M (2003). Involvement of sulfoquinovosyl diacylglycerol in the structural integrity and heat-tolerance of photosystem II. Planta.

[CR50] Siegenthaler P-A (1998) Molecular organization of acyl lipids in photosynthetic membranes of higher plants. In: Paul-André S, Norio M (eds) Lipids in photosynthesis: structure, function and genetics. Dordrecht, Netherland: Springer, p 119–144

[CR51] Su X, Ma J, Wei X, Cao P, Zhu D, Chang W, Liu Z, Zhang X, Li M (2017). Structure and assembly mechanism of plant C_2_S_2_M_2_-type PSII–LHCII supercomplex. Science.

[CR52] Umena Y, Kawakami K, Shen JR, Kamiya N (2011). Crystal structure of oxygen-evolving photosystem II at a resolution of 1.9 Å. Nature.

[CR53] Wada H, Murata N (2007). The essential role of phosphatidylglycerol in photosynthesis. Photosynth Res.

[CR54] Wei X, Su X, Cao P, Liu X, Chang W, Li M, Zhang X, Liu Z (2016). Structure of spinach photosystem II–LHCII supercomplex at 3.2 Å resolution. Nature.

[CR55] Zhang J, Ma J, Liu D, Qin S, Sun S, Zhao J, Sui SF (2017). Structure of phycobilisome from the red alga *Griffithsia pacifica*. Nature.

[CR56] Zhou F, Liu S, Hu Z, Kuang T, Paulsen H, Yang C (2009). Effect of monogalactosyldiacylglycerol on the interaction between photosystem II core complex and its antenna complexes in liposomes of thylakoid lipids. Photosynth Res.

